# Editorial: New Mind-Body Interventions That Balance Human Psychoneuroimmunology

**DOI:** 10.3389/fpsyg.2021.706584

**Published:** 2021-08-20

**Authors:** Pádraic J. Dunne, Christian Schubert

**Affiliations:** ^1^Centre for Positive Psychology and Health, Royal College of Surgeons in Ireland University of Medicine and Health Sciences, Dublin, Ireland; ^2^Clinical Department of Medical Psychology, Medical University of Innsbruck, Innsbruck, Austria

**Keywords:** psychoneuroimmunology, eating disorders, rheumatoid arthritis, psychopathology, lifestyle medicine, yoga, Pilates

As researchers, educators, practitioners, and citizens, we are collectively witnessing the rekindling of once lost, as well as novel fields of science, medicine, health and well-being that include functional, integrative and lifestyle medicine, as well as the re-invigorated subject of psychoneuroimmunology (among others). Advances in laboratory-based technologies, alongside novel social, environmental and psychology-based theories have combined to support researchers and clinicians in developing a re-established foundation for what we might call whole person, integrated health. These approaches to research, as well as prevention and treatment of disease that allow individuals to flourish, sometimes in spite of ongoing suffering, represent the future of health and well-being, as we approach the end to the first quarter of the 21st Century.

The importance of diet, exercise, sleep, substance control, cultivating healthy relationships, as well as meaning and purpose in life, to maintaining the well-being of individuals and communities, has never been more apparent (Smirmaul et al., 2020). It is equally important to understand the mechanisms behind the disturbances between these described interfaces, as it is to develop efficient, and cost-effective interventions for re-establishing homeostasis and preventing disease development.

Interventions previously described as complementary or alternative to conventional approaches to healthcare, such as yoga, meditation-based interventions and culinary approaches to health (e.g., nutriceuticals, psychobiotics, prebiotics, and anti-inflammatory diets) (Ricker and Haas, 2017; Zhong et al., 2017; Cryan et al., 2019) are now fast becoming real options for preventing non-communicable diseases or re-establishing homeostasis between our biopsychosocial interfaces. However, more quality, evidence-based research is required as we move forward.

It is evident from the themes discussed in this topic that human evolutionary development is colliding with 21st century living, to create disturbances among biological, psychological, social and environmental interfaces. The result is often the development of chronic disease states with biopsychosocial etiology. This is certainly apparent in the hypothesis and theory article by Rantala et al., who discuss the impact of proximate mechanisms and ultimate evolutionary fitness benefits that they believe, lie at the root of eating disorders. They describe the *mismatch hypothesis* of Anorexia Nervosa as one possible explanation for the development of this disorder among citizens of contemporary nations. Rantala et al., suggest that whereby “the novel situation in which previously co-adapted psychological mechanisms of food intake and mating become antagonistic. This antagonism creates a situation in which an individual is torn between opposing incentives: food rewards and mating rewards” (Rantala et al.).

The authors go on to describe eating disorders as a continuum experienced by the individual, beginning with binge eating disorder (BED) and ending with anorexia nervosa (AN); bulimia nervosa (BN) has been placed in the centre of this continuum. Rantala et al. suggest that a number of factors might explain the nature of this continuum and why individuals experience different symptoms. Pertinent to this issue, these factors include the presence of active neuroinflammation, chronic metaflammation, stress hormone production, stress resilience, disturbed serotonin levels and interestingly, the composition of the gut microflora.

Whether neuroinflammation, represented by increased plasma Interleukin (IL)-15 and chronic metaflammation, associated with elevated levels of IL-6 and Tumor Necrosis Factor (TNF)-α in peripheral blood, are part of a causal impact of eating disorders or exemplify an artifact of stress and negative thinking cycles, remain to be determined. Rantala et al. suggest supplementing diets with micronutrients and vitamins, including Zinc and Vitamin D, the latter of which has potent immunomodulatory impacts (Fletcher et al., 2012).

Staying with eating disorders (specifically AN), Martínez-Sánchez et al. describe the positive impact of a 10-week Pilates programme on psychopathology, body image disturbance and quality of life among a cohort of female children and adolescents with AN. Equally relevant to enhancing our understanding of the fundamental etiology of psychoneuroimmune-related disease processes, is the development of cost-effective, evidence-based interventions. If evolutionary-prompted, disturbed thinking processes and stress can lead to the development of psychoneuroimmune-related disease, then it is important to find interventions that help mitigate these causative factors.

It would seem that negative impulses, thinking cycles and subsequent stress responses can be alleviated with established physical exercise regimens such as Pilates. However, it remains unclear whether these and similar interventions improve quality of life by limiting inflammation, associated stress markers such as cortisol or by improving the sense of self and helping the individual to disengage from negative thinking cycles.

Adding further to our understanding of evidence—based interventions in psychoneuroimmune-related disease, Gautam et al. have studied the impact of a yoga-based lifestyle intervention on the inflammatory processes associated with Rheumatoid Arthritis. Along with a decrease at the messenger level of the classic pro-inflammatory markers (IL-6 and TNF-α), they describe a corresponding increase in the regulatory cytokine Transforming Growth Factor (TGF)-β, among intervention group participants. Furthermore, the same researchers noted that the described yoga-based lifestyle intervention was also associated with elevated levels of what they term mind-body communicative markers, including Brain-derived Neurotrophic Factor (BDNF), Dehydroepiandrostendione (DHEAS), β-endorphins, and sirtuin-1. It is important to increase the bank of biomarkers that can be used to confirm the efficacy of both pharmacological and non-pharmacological therapeutic interventions in psychoneuroimmune-related disease.

Also in this topic, Singer et al. have highlighted the often perplexing and frustrating heterogeneity of research findings in relation to both pro-inflammatory and anti-inflammatory agents at the protein and messenger level (Singer et al.). Issues with standardization of research design, poor consistency of applying non-pharmacological interventions, daily fluctuations in biomarkers, all occurring among a complex biopsychosocial reality, add to disparities in published observations. The pleiotropic nature of potent cytokines such as IL-6 is well-described in this case; as is the importance of suggestive meaning in response to complementary and alternative interventions as a part of complex psychosomatic mechanisms.

Singer et al's integrative single-case design also demonstrates the importance and depth of information that can be garnered from this type of investigation; the randomized controlled trial is not the only means of acquiring an evidence base in this field.

Although great strides have been made in this re-emerging field, a number of outstanding questions remain. Which comes first? Disturbances among psychoneuroimmune interfaces or psychological issues, related to social and environmental dysfunction? The answers will guide the development of the most efficacious therapeutic interventions. It is tempting to trial existing, approved anti-inflammatory therapeutic agents for psychopathology. However, with this approach we run the risk of treating an artifact of a larger more complicated disease process, rather than the root cause. Regardless, the immune system remains an important player in the health of humans and not just in a classic context of protection against microbial infection. Enhancing our understanding of the role of immunological factors among the greater biopsychosocial sphere, will help us develop novel strategies to maintain and sustain whole person health in the future.

## Author Contributions

PJD, JR, and CS contributed to the design of the topic and review of approved manuscript. PJD and CS contributed to this article and approved the submitted version.

## Conflict of Interest

The authors declare that the research was conducted in the absence of any commercial or financial relationships that could be construed as a potential conflict of interest.

## Publisher's Note

All claims expressed in this article are solely those of the authors and do not necessarily represent those of their affiliated organizations, or those of the publisher, the editors and the reviewers. Any product that may be evaluated in this article, or claim that may be made by its manufacturer, is not guaranteed or endorsed by the publisher.
